# Better and Worse: A Dual-Process Model of the Relationship between Core Self-evaluation and Work-Family Conflict

**DOI:** 10.3389/fpsyg.2016.01579

**Published:** 2016-10-13

**Authors:** Kun Yu

**Affiliations:** School of Labor and Human Resources, Renmin University of ChinaBeijing, China

**Keywords:** core self-evaluation, work stress, career resilience, work-family conflict

## Abstract

Based on both resource allocation theory (Becker, 1965; Bergeron, 2007) and role theory (Katz and Kahn, 1978), the current study aims to uncover the relationship between core self-evaluation (CSE) and three dimensions of work interference with family (WIF). A dual-process model was proposed, in which both work stress and career resilience mediate the CSE-WIF relationship. The mediation model was tested with a sample of employees from various organizations (*N* = 561). The results first showed that CSE was negatively related to time-based and strain-based WIF and positively related to behavior-based WIF via the mediation of work stress. Moreover, CSE was positively associated with behavior-based and strain-based WIF via the mediation of career resilience, suggesting that CSE may also have its “dark-side.”

## Introduction

Work and family consist the two most important realms for most adults (Andrews and Withey, 1976), and the incompatibility of role pressures between these two realms is known as work-family conflict (Greenhaus and Beutell, 1985). Work-family conflict was considered a bidirectional construct, including work interference with family (WIF) and family interference with work (FIW; Frone et al., 1992a). Since WIF is more prevalent than FIW and has a more severe impact on family life than that FIW has on work domain (Frone et al., 1992b; Frone, 2003), how work interferes with family has been the most primary concern of work-family conflict (Greenhaus and Beutell, 1985) and received a majority of research focus in recent years (Frone et al., 1992b). WIF was associated with various unfavorable consequences, such as job dissatisfaction (Bedeian et al., 1988; Bacharach et al., 1991), burnout (Pleck et al., 1980), turnover intention (Burke, 1988), low well-being (Matthews et al., 2014; Goh et al., 2015), and depression (Frone et al., 1992b).

As the destructive outcomes that work-family conflict brings to individuals’ work and life, considerable attentions has been gained on the causes of work-family conflict, especially work interference with family (WIF), in past decades (Eby et al., 2005). Situational factors such as work stressors (Frone et al., 1992a; Goh et al., 2015), social support (Carlson and Perrewé, 1999; Nielson et al., 2001), and personal factors such as individual initiative (Bolino and Turnley, 2005) and locus of control (Andreassi and Thompson, 2007) were found to be associated with WIF.

However, although Greenhaus and Beutell (1985) theoretically grouped sources of work-family conflict into three categories, including time-based (i.e., time competition between work and family roles), strain-based (i.e., strain produced in one role affects performance in another role) and behavior-based conflict (i.e., in-role behavior of one role is not compatible with expectations of another role), most studies on the antecedents of work-family conflict only measure time-based and strain-based conflict (O’Driscoll et al., 2005). The investigation of the behavior-based work-family conflict has been largely absent from the literature (Dierdorff and Ellington, 2008).

Moreover, core self-evaluation (CSE), a latent personality construct comprised of four components, namely self-esteem (Harter, 1990), locus of control (Rotter, 1966), generalized self-efficacy (Bandura, 1982), and emotional stability (Watson, 2000), was found to be positively associated with a series of important work and life outcomes, such as performance (Erez and Judge, 2001; Grant and Sonnentag, 2010; Grant and Wrzesniewski, 2010; Rode et al., 2012), income (Judge and Hurst, 2007; Judge et al., 2009), well-being (Creed et al., 2009) and health (Judge et al., 2012). However, to date, little research has been conducted to examine the association between CSE and work-family conflict. Few exceptions (e.g., Boyar and Mosley, 2007; Haines et al., 2013) are cross-sectional studies treating work-family conflict as a unidimensional construct and is a lack of investigation of the underlying influence processes, leaving how CSE affects the three dimensions of work-family conflict unclear.

**FIGURE 1:**
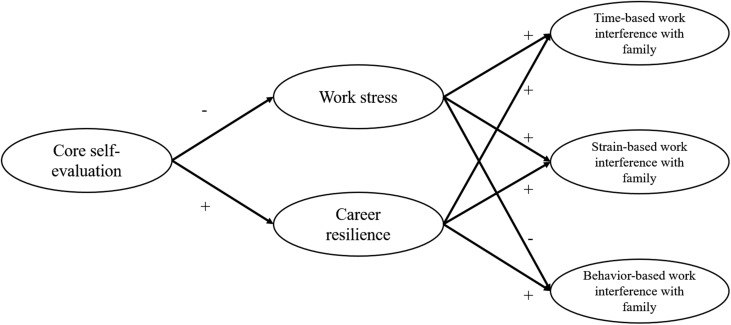
**Proposed research model.** The first proposed mediation path was from core self-evaluation to three dimensions of work interference with family via work stress. The second proposed mediation path was from core self-evaluation to three dimensions of work interference with family via career resilience.

To address this theoretical void, the current study developed and tested a dual-process theoretical framework linking CSE and time-based, strain-based, and behavior-based WIF, as can be seen in **Figure 1**. First, based on the resource allocation theory (Becker, 1965; Bergeron, 2007) which suggests that finite personal resources (e.g., time and energy) constraint individuals’ capacity to reach multiple job requirements, I proposed that work stress mediates the negative relationship between CSE and time-based and strain-based WIF, and mediates the positive relationship between CSE and behavior-based WIF. Specifically, I expected that CSE is negatively associated with work stress, and work stress is positively related to time-based and strain-based WIF, and negatively associated with behavior-based WIF. Second, based on the role theory (Katz and Kahn, 1978) which depicts that each work role has a specific pattern of in-role behavior, I proposed that career resilience, a dimension of career motivation that refers to individuals’ persistence toward career goals and resistance to unfavorable career situations, mediate the relationship between CSE and behavior-based WIF. Moreover, based on the resource allocation theory (Becker, 1965; Bergeron, 2007), I also proposed that career resilience mediates the positive relationship between CSE and time-based and strain-based WIF, and mediates the negative relationship between CSE and strain-based WIF. Specifically, I expected that CSE is positively associated with career resilience, and career resilience is positively related to behavior-based WIF and time-based WIF, while negatively associated with strain-based WIF.

### Core Self-evaluation

Core self-evaluation was originally proposed by Judge et al. (1997) as a trait predictor of job satisfaction. Consisting of self-esteem (Harter, 1990), locus of control (Rotter, 1966), generalized self-efficacy (Bandura, 1982), and emotional stability (Watson, 2000), CSE was considered to be a high-order, fundamental and positive concept of one’s value and functioning in the world (Judge et al., 1997, 2003). Since it was proposed, CSE has emerged as an important trait predictor of numerous outcomes in organizations (Johnson et al., 2008; Judge, 2009; Judge and Kammeyer-Mueller, 2011), including task performance (e.g., Erez and Judge, 2001; Kacmar et al., 2009), income (e.g., Judge and Hurst, 2007; Judge et al., 2009), occupational status (e.g., Judge and Hurst, 2008), citizenship behavior (e.g., Rich et al., 2010; Rode et al., 2012), counter-productive behavior (e.g., Ferris, 2011), satisfaction (e.g., Best et al., 2005; Srivastava et al., 2010), stress (e.g., Judge et al., 2012; Lim and Tai, 2014), LMX (e.g., Sears and Hackett, 2015), commitment (e.g., Brown et al., 2007; Stumpp et al., 2009) and job search behavior (e.g., Brown et al., 2007).

Furthermore, researchers have also brought CSE out of organizations and found that CSE also benefits outcomes in a broader life domain, including well-being (Creed et al., 2009), life satisfaction (Judge and Bono, 2005) and health (Judge et al., 2012). Moreover, there was initial evidence that CSE was linked to work-family conflict (Boyar and Mosley, 2007), suggesting that CSE has an adverse effect on WIF, which was measured as a unidimensional construct. Since it’s a lack of mechanism investigation, it was unable to determine how CSE was associated with WIF.

### CSE and WIF: The Mediating Role of Work Stress

As CSE represents an overall positive self-concept, individuals with a high level of CSE, which includes a high level of self-esteem, would think of themselves as being competent (Judge et al., 1997). Therefore, this self-appraisal of competence would let them be less influenced by external pressures, such as work stressors. In addition, the positive affect brought about by CSE may also help with the stress-coping process (Folkman, 2008) and, therefore, reduce stress. Thus, in line with previous research, I predict that:

Hypothesis 1: Core self-evaluation is negatively associated with work stress.

Based on the resource allocation theory (Becker, 1965; Bergeron, 2007), personal resources such as time and energy are finite, and resources consumed in one task or domain would reduce the resources available for other tasks or domains. For individuals who are experiencing a high level of work stress to cope efficiently, they have to expend their limited personal resources. Hence, resources such as time for the family domain would be reduced. This would bring about time-based WIF. In addition, as the definition of strain-based WIF is the negative spillover of affect from the work domain to the family domain (Greenhaus and Beutell, 1985), it is reasonable to argue that work stress is positively related to strain-based WIF. Moreover, while work stress spill over from workplace to family domain, stress will become pervasive across work and family roles (Eckenrode and Gore, 1990). Stress coping then is not a specific behavior in the workplace but a common behavior in both work and family domains. The consistency of stress coping behavior across work and family life may reduce behavior incompatibility between these two domains and bring about a low level of behavior-based work interference with family. Thus, it is hypothesized that:

Hypothesis 2: Work stress is positively associated with (a) time-based work interference with family and (b) strain-based work interference with family.
Hypothesis 3: Work stress is negatively associated with behavior-based work interference with family.

The above arguments suggest that CSE and WIF are connected, at least partially, through a work stress path. Specifically, individuals who possess a high level of CSE may experience a lower level of work stress, and the low work stress brought by high CSE may, in turn, reduce their time-based, strain-based WIF but increase behavior-based WIF. Indeed, some empirical studies have linked CSE to work stress and found that CSE was negatively associated with work stress (Brunborg, 2008; Kluemper, 2008; Judge et al., 2012; Takeuchi et al., 2015). There was also evidence that work stress is positively associated with WIF (mostly time-based and strain-based; Ford et al., 2007). Thus, combined with the above argument and evidence, it is hypothesized that:

Hypothesis 4: Work stress mediates the negative relationship between core self-evaluation and (a) time-based work interference with family and (b) strain-based work interference with family.
Hypothesis 5: Work stress mediates the positive relationship between core self-evaluation and behavior-based work interference with family.

### CSE and WIF: The Mediating Role of Career Resilience

Career resilience is a sub-dimension of career motivation, which includes individual traits and related career decisions and behaviors that manifest individuals’ career identity, career insight, and career resilience (London, 1983). Career resilience demonstrates individuals’ initiative and maintains performance level, especially when facing negative work situations (Noe et al., 1990). For employees with a high level of CSE, they will not only more likely be satisfied by their work (e.g., Best et al., 2005; Brown et al., 2007), but also more likely engage in their work (Rich et al., 2010), stick to their self-concordant goals (Judge and Bono, 2005) and have stronger work motivation (Ferris, 2011). Therefore, career resilience, as a sub-dimension of career motivation, is also expected to be high for individuals with a high level of CSE. Thus, it is hypothesized that:

Hypothesis 6: Core self-evaluation is positively associated with career resilience.

According to the role theory (Katz and Kahn, 1978), employees engage specific patterns of behavior (i.e., in-role behavior) in their specific work roles. While career resilience helps individuals cope with adverse work environments and keep moving toward to their career goals, it calls for specific in-role behaviors from the individuals, such as setting difficult goals, taking extra working time, and finding better ways to do the job (Noe et al., 1990). These task-oriented and hard-driving behaviors, though they benefit individuals’ work-related outcomes, may not be compatible with the expectation of the family role, in which being supportive and loving may be more favorable (O’Driscoll et al., 2005). This would lead to a high level of behavior-based WIF, which is an indicator of incompatible behaviors in the work and family domains.

Moreover, like similar findings that citizenship behavior was associated with role overload (Bolino and Turnley, 2005), the hard-driving resilience behavior may cause overload as well. As role overload is considered a component of role stressors (Eatough et al., 2011), it is reasonable that resilience would also lead to role-related strain and then spill over into the family domain, which is indicated by a high level of strain-based WIF. In addition, based on the resource allocation theory (Becker, 1965; Bergeron, 2007), hard-driving behaviors associated with career resilience will consume individuals’ finite resources, such as time. Therefore, individuals’ resources for family life would be reduced accordingly, which may bring about time-based WIF. Thus, it is hypothesized that:

Hypothesis 7: Career resilience is positively associated with (a) time-based work interference with family, (b) strain-based work interference with family and (c) behavior-based work interference with family.

Along with the above reasoning, CSE and WIF may also be linked through a career resilience path. Specifically, individuals with a high level of CSE would be more likely to have a high level of career resilience, producing task-oriented and hard-driving behavior. This type of behavior is not only incompatible with the family role and leads to a high level of behavior-based WIF, but also resources consuming and results in a high level of time-based WIF. This argument could be backed up by findings revealing the negative effect of some “positive” construct, such as citizenship behavior, on work-family relationships (Bolino and Turnley, 2005). Moreover, CSE may also be negatively associated with strain-based WIF through the mediation of career resilience, as which would keep individuals away from stressors’ influence (Bergeman and Deboeck, 2010). Thus, it is hypothesized that:

Hypothesis 8: Career resilience mediates the positive relationship between core self-evaluation and (a) time-based work interference with family, (b) strain-based work interference with family and (c) behavior-based work interference with family.

## Materials and Methods

### Participants and Procedure

The data of this study were collected as part of a large and publicly available dataset called The Professional Worker Career Experience Survey (PWCES^1^). All potential respondents were contacted via email, and a total of 752 professional employees from various organizations across the central United States responded to the survey. Among the 752 participants, 561 (response rate = 74.60%) have completed the answer on the study variables (i.e., core self-evaluation, work stress, career resilience, work interference with family and control variables) of the current research. The average age of the 561 subjects is 31.92 (*SD* = 9.75), 56.3% of them were male, 91.2% have bachelor’s degree or above, and reported the following ethnicities: 91.8% Caucasian, 2.1% African–American, 3.4% Asian, and 2.7% Other. Among all 561 participants, 38.7% were employed in business and financial occupations, 35.1% in computer and mathematical occupations, and 26.2% in a variety of other occupations.

### Measures

#### Core Self-evaluation

Core self-evaluation was measured by Judge et al.’s (2003) 12-item core self-evaluation scale, a unidimensional scale to assess one’s core evaluation about oneself. Core self-evaluation consists of four personality traits, namely generalized self-efficacy, self-esteem, locus of control, and emotional stability (Judge et al., 2003). Participants were asked to rate their agreement with the descriptions of self-evaluation on a 5-point Likert scale (from 1 = strongly disagree to 5 = strongly agree). A sample item is “I am confident I get the success I deserve in life.” The Cronbach’s α of the scale was 0.84.

#### Work Stress

Work stress was measured using Lait and Wallace’s (2002) 6-item work stress scale, which was design to evaluate employees’ perception of stress while not confounding with other related factors or outcomes. Participants were asked to rate their agreement with the descriptions on a 6-point Likert scale (from 1 = strongly disagree to 6 = strongly agree). A sample item is “I feel frustrated with my work.” The Cronbach’s α of the scale was 0.91.

#### Career Resilience

Career resilience was measured by 13-item career resilience dimension of Noe et al.’s (1990) career motivation scale, which is a three-dimension scale (career resilience, career identity and career insight) assessing individuals’ motivations in career decisions and career success. Participants were asked to rate their agreement with the statements about their resilience at work on a 6-point Likert scale (from 1 = strongly disagree to 6 = strongly agree). A sample item is “I accept compliments rather than discount them.” The Cronbach’s α of the scale was 0.83.

#### Work Interference with Family

Work interference with family was measured by three work interference with family dimensions of Carlson et al.’s (2000) work-family conflict scale. Each dimension has three items. Participants were asked to rate their agreement with the statements about how their work affect family life on a 6-point Likert scale (from 1 = strongly disagree to 6 = strongly agree). A sample item of the time-based work interference with family (WIFT) is “My work keeps me from my family activities more than I would like.” A sample item of the strain-based work interference with family (WIFS) is “I am often so emotionally drained when I get home from work that it prevents me from contributing to my family.” Moreover, a sample item of the behavior-based work interference with family (WIFB) is “Behavior that is effective and necessary for me at work would be counterproductive at home.” The Cronbach’s α for time-based, strain-based, and behavior-based WIF was 0.84, 0.87, and 0.80, respectively.

#### Control Variables

Participants’ gender, age, educational level was controlled in the current study as previous research has revealed their influence to work-family conflict (Michel et al., 2011). Besides, as negative affectivity is considered to systematically influence respondents’ rating on self-report questionnaires and brings common method bias (Podsakoff et al., 2003), we controlled neuroticism, which is seen equivalent with negative affectivity (Burke et al., 1993), in the current study too. Neuroticism was measured with the 12-item neuroticism dimension of the NEO Five-Factor Inventory (NEO-FF-I; Costa and McCrea, 1992). Participants were asked to rate their agreement on a 5-point Likert scale (from 1 = strongly disagree to 5 = strongly agree). A sample item is “I often feel tense and jittery.” The Cronbach’s α of the neuroticism scale was 0.88.

## Results

The means, standard deviations, reliabilities, and correlations among the study variables are presented in **Table 1**. CSE was negatively associated with stress (*r* = -0.57, *p* < 0.01) and positively associated with career resilience (*r* = 0.50, *p* < 0.01). Moreover, CSE was negatively associated with time-based work interference with family (WIFT; *r* = -0.20, *p* < 0.01) and strain-based work interference with family (WIFS; *r* = -0.46, *p* < 0.01), while positively associated with behavior-based work interference with family (WIFB; *r* = 0.21, *p* < 0.01). Furthermore, Work stress was positively associated with WIFT (*r* = 0.32, *p* < 0.01) and WIFS (*r* = 0.55, *p* < 0.01) and negatively associated with WIFB (*r* = -0.21, *p* < 0.01); and on the contrary, career resilience was positively associated with WIFB (*r* = 0.21, *p* < 0.01) and negatively associated with WIFS (*r* = -0.15, *p* < 0.01). With respect to control variables, gender was positively correlated with WIFS (*r* = 0.13, *p* < 0.01) and WIFB (*r* = 0.14, *p* < 0.01), and age was positively correlated with WIFB (*r* = 0.19, *p* < 0.01), while educational level positively correlated with WIFS (*r* = 0.12, *p* < 0.01). Thus, gender, age, and educational level were controlled in the further analysis.

**Table 1:** Descriptive statistics for study variables.

		*M*	*SD*	1	2	3	4	5	6	7	8	9	10
(1)	Gender	0.44	0.50	-									
(2)	Age	39.12	9.76	0.01	-								
(3)	Education	6.41	1.10	-0.14ˆ**	0.04	-							
(4)	Neuroticism	2.43	0.68	0.13ˆ**	-0.05	-0.07	(0.88)						
(5)	CSE	3.74	0.54	-0.02	-0.06	0.06	-0.80ˆ**	(0.84)					
(6)	Work stress	2.50	1.24	0.06	0.00	-0.04	0.51ˆ**	-0.57ˆ**	(0.91)				
(7)	Career resilience	4.83	0.60	0.08ˆ*	0.04	0.05	-0.41ˆ**	0.50ˆ**	-0.27ˆ**	(0.83)			
(8)	WIFT	3.04	1.29	-0.06	0.02	0.05	0.15ˆ**	-0.20ˆ**	0.32ˆ**	-0.04	(0.84)		
(9)	WIFS	2.91	1.26	0.13ˆ**	0.05	0.12ˆ**	0.45ˆ**	-0.46ˆ**	0.55ˆ**	-0.15ˆ**	0.53ˆ**	(0.87)	
(10)	WIFB	4.04	0.97	0.14ˆ**	0.19ˆ**	0.01	-0.15ˆ**	0.21ˆ**	-0.21ˆ**	0.21ˆ**	-0.05	-0.14ˆ**	(0.80)

*N = 561. Gender was coded “0” for men and “1” for women. Coefficient alphas are reported in parentheses along the diagonal. CSE, core self-evaluation; WIFT, time-based work interference with family; WIFS, strain-based work interference with family; WIFB, behavior-based work interference with family. ^∗^p < 0.05; ^∗∗^p < 0.01(two-tailed test).*

### Tests of Measurement Model

To examine whether the constructs measured in the current research are distinguishable from each other, we conducted a confirmatory factor analysis (CFA) using Mplus 7 (Muthén and Muthén, 1998–2012). Specifically, the measurement model included all key constructs and covariates (i.e., CSE, work stress, career resilience, WIFT, WIFS, WIFB and neuroticism). We created four parcels of items for CSE, career resilience and neuroticism using random assignment procedure (Little et al., 2002) to improve the sample size to parameter ratio, which would magnify standard errors and adversely affect stability of the estimate (Landis et al., 2000). Specifically, both CSE and neuroticism had four parcels and each parcel included three items. Career resilience had four parcels and each parcel included 3–4 items.

CFA results for the measurement model indicated that the seven-factor measurement model (i.e., all variables are independent of each other) fits the data well, χ^2^(303) = 1098.45, *p* < 0.01, Comparative Fit Index (CFI) = 0.92, Tucker-Lewis Index (TLI) = 0.90, Root Mean Square Error of Approximation (RMSEA) = 0.07. All items/ indicators loaded significantly (*p* < 0.01) on their corresponding factor with standardized factor loadings ranging from 0.58 to 0.92. This measurement model fits the data better than all 21 constrained models in which any two of the seven latent factors were combined [1162.67 ≤ Δχ^2^(Δ*df* = 6) ≤ 2205.61, *p* < 0.01]. These results provided support for the constructs distinctiveness of our measurement model.

### Tests of Two-Path Mediation Model

Using Mplus 7, we tested a two-path mediation model (**Figure 1**) on the basis of the measurement model established above. First, work stress and career resilience were both predicted by the core self-evaluation. Second, WIFT, WIFS were predicted by work stress and WIFB was predicted by career resilience. Third, we controlled for the effects of gender, age, educational level, and neuroticism in all analyses.

**FIGURE 2:**
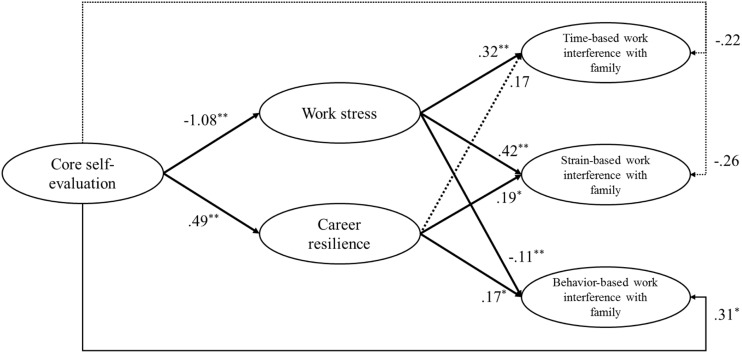
**Research path model.** *N* = 561. ^∗^*p* < 0.05; ^∗∗^*p* < 0.01(two-tailed test).

As can be seen from **Table 2** and **Figure 2**, in this full-mediation model, the direct paths from CSE to work stress (β = -0.47, *p* < 0.01) was significantly negative and to career resilience (β = 0.44, *p* < 0.01) significantly positive. Thus, Hypothesis 1 and Hypothesis 6 were supported. In addition, the direct paths from work stress to WIFT (β = 0.34, *p* < 0.01) and WIFS (β = 0.44, *p* < 0.01) were significantly positive, and to WIFB was significantly negative (β = -0.11, *p* < 0.01). Thus, Hypothesis 2 and Hypothesis 3 were supported. Moreover, the direct path from career resilience to WIFB (β = 0.17, *p* < 0.05) and WIFS (β = 0.19, *p* < 0.05) were significantly positive, while the direction path to WIFT was not significant (β = 0.17, *p* > 0.05). Thus, Hypothesis 7b and Hypothesis 7c were supported, and Hypothesis 7a was not supported.

**Table 2:** Direct and indirect effect and the associated 95% confidence intervals.

Predictor	Mediator		Dependent variable					
	Work Stress	Career resilience	WIFT		WIFS		WIFB	
			Direct	Indirect	Direct	Indirect	Direct	Indirect
Gender	0.08	0.13^∗∗^	-0.20		0.17		0.26^∗∗^
Age	0.00	0.00	0.00		0.01		0.02^∗∗^	
Education	0.01	0.02	0.06		-0.10^∗^		0.00	
Neuroticism	0.24^∗^	-0.06	-0.07		0.33^∗∗^		0.15	
CSE	-1.08^∗∗^	0.49^∗∗^	-0.22		-0.26		0.31^∗^	
Stress			0.32^∗∗^	-0.35^∗∗^ (-0.49,-0.22)	0.42^∗∗^	-0.45^∗∗^ (-0.62, -0.31)	-0.11^∗∗^	0.12^∗^ (0.04,0.23)
Resilience			0.17	0.08 (-0.01,0.21)	0.19^∗^	0.09^∗^ (0.02, 0.21)	0.17^∗^	0.09^∗^ (0.02,0.18)

*N = 561. 95% Confidence intervals of indirect effect were calculated from the bootstrapping analyses using the bias corrected method and are reported in parentheses. CSE, core self-evaluation; WIFT, time-based work interference with family; WIFS, strain-based work interference with family; WIFB, behavior-based work interference with family. ^∗^p < 0.05; ^∗∗^p < 0.01(two-tailed test).*

We then used a bootstrap approach to test the mediating effect of work stress in the relationship between CSE and WIFT and WIFS, and the mediating effect of career resilience in the relationship between CSE and WIFB. On the basis of a resampling size of 1,000, the bootstrap result demonstrated that both indirect effects of CSE on WIFT, WIFS and WIFB via work stress were significant (for WIFT, β = -0.35, *p* < 0.01, 95% CI = [-0.49, -0.21]; for WIFS, β = -0.45, *p* < 0.01, 95% CI = [-0.60, -0.30]; and for WIFB, β = 0.12, *p* < 0.05, 95% CI = [0.04, 0.23]). Thus, Hypothesis 4 and Hypothesis 5 were supported. Moreover, the indirect effects of CSE on WIFB and WIFS through career resilience were significant too (for WIFB, β = 0.09, *p* < 0.05, 95% CI = [0.02, 0.18]; for WIFS, β = 0.09, *p* < 0.05, 95% CI = [0.02, 0.21]), while the indirect path from CSE to WIFT via career resilience was not significant (β = 0.08, *p* > 0.05). Thus, Hypothesis 8b and Hypothesis 8c were supported, and Hypothesis 8a were not supported.

## Discussion

### Theoretical Implications

Based on the resource allocation theory (Becker, 1965; Bergeron, 2007) and role theory (Katz and Kahn, 1978), the present research proposed a dual-process model of the relationship between CSE and time-based, strain-based, and behavior-based dimensions of WIF with work stress and career resilience as mediators. The results showed that work stress mediated the negative relationship between CSE and time-based/ strain-based WIF, and mediated the positive relationship between CSE and behavior-based WIF. Moreover, career resilience mediated the positive relationship between CSE and behavior-based/ strain-based WIF. By the investigation of this integrated mediation model, the present study advances current understandings of both CSE and work-family literature.

First, while a major proportion of previous research was based on the measurement of time-based and/or strain–based WIF (O’Driscoll et al., 2005), evidence of the nature and antecedents of work-family conflict were, therefore, biased and incomplete. In the present study, we incorporated behavior-based WIF, which was largely neglected in previous investigations (Dierdorff and Ellington, 2008), with time-based and strain-based WIF, and conducted an integrated framework of work-family conflict. On one hand, consistent with the argument of the resource allocation theory (Becker, 1965; Bergeron, 2007), CSE was negatively associated with time-based and strain-based WIF and positively associated with behavior-based WIF via the mediation of work stress. On the other hand, consistent with role theory (Katz and Kahn, 1978), CSE was positively associated with strain-based and behavior-based WIF via the mediation of career resilience. These findings uncovered the complex relationship between CSE and WIF. The investigation of the antecedents, such as CSE, of the integrated construct of work-family conflict could provide a more comprehensive picture of how individual traits are associated with work-family conflict.

Second, findings of the current study contribute to the literature of resource allocation theory. Previous research has applied resource allocation framework into situations such as attention allocation in task performance (Hockey, 1997), time allocation between in-role and extra-role behavior (Bergeron, 2007), or resource allocation in group performance (Nielsen et al., 2012). Grawitch et al. (2010) conducted a theoretical framework of resource allocation within the work-life interface and proposed individual traits as key factors to affect the resource allocation. In the current study, I adopted the resource allocation framework (Becker, 1965; Bergeron, 2007) and proposed the relationship between CSE and WIF is mediated by work stress. By empirically demonstrating the mediating effect of work stress on the relationship between CSE and three dimensions of WIF, the current research extended the resource allocation framework to the area of work-family interface and increased the generalizability of the framework.

Third, the present research revealed that CSE was positively associated with strain-based WIF via the mediation of career resilience, and was positively associated with behavior-based WIF via the mediation of both work stress and career resilience. This detrimental effect of CSE on family domains is consistent with recent findings on the “dark-side” of positive behavior, such as prosocial behavior (Bolino and Grant, 2016), citizenship behavior (Bolino and Turnley, 2005; Bolino et al., 2013), and leadership behavior (Liu et al., 2012). CSE was previously thought to be a positive construct (Judge et al., 1997), and little if any research considers its possible negative effect on individual or organizational outcomes. The current examination of the possible “dark-side” of CSE provides a new route to better understanding the nature and influence of CSE.

### Limitations, Future Directions, and Practical Implications

The present study has some limitations. First, the cause-and-effect conclusion about the impact of CSE on three dimensions of work-family conflict through work stress and career resilience cannot be reached based on the cross-sectional design of the current study. Longitudinal studies are urged to demonstrate the predictive validity of CSE, and the sequence of the two mediation paths. Second, although I tested the mediation mechanisms of the relationship between CSE and work-family conflict, the boundary conditions on which the relationship will still be valid or no longer be significant were a lack of investigation. Future research could address this point, explore possible moderators in this relationship, and help us better understand the role of CSE across work and family domains. For instance, abusive supervision may weaken the path between CSE and work-family conflict via work stress, as abusive supervision may result in a high level of stress for all employees (Wu and Hu, 2009).

The findings of the current research would provide some practical implications. First, for employees working in organizations, CSE was considered a “good” trait that brings numerous favorable job-related outcomes, in such areas as income (e.g., Judge and Hurst, 2007), performance (e.g., Erez and Judge, 2001), and job satisfaction (e.g., Best et al., 2005). However, cautions should be given to high-CSE employees. They may experience unfavorable consequences in their family domains because the in-role behaviors they act out within the organization are not suitable for family life. This suggests that individuals have to switch effectively from in-role behavior in the work domain to in-role behavior in the family domain, in particular for those who are high on CSE. Second, and similarly, although career resilience is helpful when confronting situational constraints in the workplace (Noe et al., 1990), individuals should be conscious of the possible adverse impact of career resilience on their family life, as a high level of career resilience could lead to both strain-based and behavior-based work interference with family.

## Conclusion

In summary, the findings of the present study suggest that CSE has a complicated relationship with work-family conflict. On one hand, CSE was negatively associated with time-based and strain-based work interference with family via the mediation of work stress. On the other hand, CSE was positively associated with behavior-based and strain-based work interference with family via the mediation of career resilience. Taken together, these findings extend previous research on the role of CSE in and out of organizations by exploring its “dark-side” as well as its “bright-side” in work-family relationships. This research shed some new light on a more comprehensive understanding of both CSE and work-family conflict.

## Author Contributions

The author confirms being the sole contributor of this work and approved it for publication.

## Conflict of Interest Statement

The author declares that the research was conducted in the absence of any commercial or financial relationships that could be construed as a potential conflict of interest.

## Footnotes

1 http://www.icpsr.umich.edu/icpsrweb/ICPSR/studies/26782
